# American Medical Association

**Published:** 1888-06

**Authors:** 


### American Medical Association.

The thirty-ninth annual meeting of the American Medical Association was held in Cincinnati, May 8th to 11th, inclusive. There was a very large attendance and many new members were admitted. The meeting was eminently successful. More work had been prepared for the various sections, and indeed for the general meetings, than could possibly be utilized. Many papers were read by title for want of time. The section on Dental and Oral Surgery was not as largely attended as we could have wished, but its meetings were very profitable and several most excellent papers were read and discussions upon them were of a high order. As evidence of this fact we may invite the attention of the reader to the first article in the present number of the REGISTER from the pen of Dr. Brittan, of Detroit, whose writings are always worthy of careful consideration and study. This section would undoubtedly have been far more largly attended had it not been that three or four leading dental societies were in session about that time.

The fears that have been expressed about the reception to membership, of those holding the degree of D.D.S. proved to be wholly without foundation, as those holding this degree and being delegates from reputable dental societies were admitted to membership without question upon the presentation of their credentials. We never entertained a doubt as to the course that would be pursued after the unanimous adoption of the Resolution pertaining to dentists at Chicago, in 1887. This action together with that which has been done hitherto, settles the status of dentistry without regard to what may be said by individuals here or there.

